# Is family-friendly policy (FFP) working in the private sector of South Korea?

**DOI:** 10.1186/2193-1801-2-561

**Published:** 2013-10-25

**Authors:** Young-Hee Kang

**Affiliations:** Latin American Regional Studies Team, Korea University, 119H Central Square, 145 Anam-ro, seongbuk-gu, Seoul, 136-701 Republic of Korea; Korean Academy of Organization and Management Meeting, Daejeon, South Korea

**Keywords:** Family-friendly policies, Job satisfaction, Organizational commitment, South Korea, The private sector

## Abstract

Using the Korean Labor and Income Panel Study (KLIPS), I investigated the impact of family-friendly policies (FFPs) on job satisfaction and organizational commitment in the private sector of South Korea. Paid leave, childcare leave, and support for housing are positively related to both job satisfaction and organizational commitment. Sick leave is positively related to organizational commitment. However, subsidized family event cost is a marginally significant predictor of job satisfaction and organizational commitment. In addition, the relationships between subsidized childcare cost and employee attitudes were not supported. Implications and suggestions for future research are discussed.

## Introduction

A dynamic workforce change has contributed to making ‘family-friendly policies (FFPs)’ an important issue in human resource management (Moon and Roh 2010). The proportion of female workers in the workforce has increased, which in turn has caused the number of male workers with working spouses to rise. As the number of married women who are struggling with childcare has increased, maintaining balance between work and family life has become a critical challenge at both the individual and the societal levels. Along these lines, much research has focused on providing evidence that FFPs have positive effects on employees’ productivity and attitudes towards their job or organizations (Bashir and Ramay 2008). In particular, the literature has discussed the impact of FFPs on job satisfaction and organizational commitment (e.g., Chiu and Ng 1999; Cho and Lee 2001; Park and Kim 2001; Pottas et al. 2007).

Research on the relationships between FFPs and both job satisfaction and organizational commitment has flourished in the U.S. However, as national contexts or cultures affect human resource management (HRM) practices (Gooderham et al 1998; Huo et al. 2002), FFPs may vary across countries in that they include leave programs and some allowances. There are some universal FFPs such as paid leave, childcare leave, sick leave, parental leave, and maternity leave, which are reported in the literature on FFPs (e.g., Chiu and Ng 1999; Grover and Crooker 1995; Moon and Roh 2010). In addition to leave programs, there are some family-friendly programs unique to South Korea, such as partial payment of family events (wedding, funerals, etc.) and support for housing.

National work environments and pay systems may affect the relationships between the availability of family-friendly policies and employee attitudes. For example, as employee pay consists of base pay related to the task and year-end incentive bonus in the U.S., except for the base pay, there is no extra bonus such as family allowance (Ahn 1999: p.33). However, in South Korea which has been influenced by Confucianism, harmony, cooperation, and mutual help have been considered virtues. Such Confucian values are also reflected in Korean HRM practices such as remuneration and performance evaluation (Bae and Rowley 2001). For instance, in addition to salaries, employers provide their employees with benefits which aim to secure the standard of living of employees and their families in South Korea (Kim 1996: p. 392). Such benefits are routinely part of the pay in South Korea (Ahn, 1999; Kim et al. 2004). National differences in the pay may influence the extent to which the family-friendly policies make effects on job satisfaction and organizational commitment. Therefore, it is important to understand the relationships between family-friendly policies and both job satisfaction and organizational commitment in contexts outside the U.S./West.

Also, little research on FFPs has been conducted on employees working in the private sector of South Korea, while considerable research on the impact of FFPs on employee attitudes has been conducted in the public sector (e.g., Moon and Roh 2010; Park and Kim 2001). There may be systematic differences between the private and the public sector. For example, compared with the private sector, the public sector is likely to secure regular work hours for employees with less unpaid over-time (Moon and Roh 2010). Also, the leave programs and benefits of the public sector are likely to be governed by the National Official Law, the Local Official Law, or the Educational Official Law.

Thus, in this study I examined the relationship between the availability of FFPs and job satisfaction in the private sector of South Korean contexts. Organizational support theory suggests that employee attitude toward their job can be influenced by the extent to which they feel organizational support for their socioeconomic needs (Eisenberger et al. 1986). This implies that the availability of FFPs may contribute to job satisfaction because workers are likely to perceive it as organizational support. I also hypothesized that the availability of FFPs has positive effects on organizational commitment. According to social exchange theory, employees may try to be more committed to their companies in order to reciprocate FFPs. Even though research has been mainly conducted on FFPs in the U.S. context and the public sector of South Korea, this study is one of the few that focuses on the private sector of South Korea.

In the pages that follow, I discuss the characteristics of the FFPs in South Korea. Following this, I provide theoretical backgrounds for relationships between the availability of FFPs and job satisfaction as well as organizational commitment. I then examine these hypothesized relations using the Korean Labor and Income Panel Study (KLIPS). Also, implications and suggestions for future research are discussed.

### Family-friendly benefits in South Korea

In South Korea, employees are entitled to take paid leaves of 15-25 days depending on the number of years they have worked in their current organization (the Labor Standard Act^a^). According to ‘Equal Employment and Work-Life Balance Protection Act’, in cases employees need to take care of their family who are sick due to accident and old age, employees can take sick leaves. Childcare leaves can be taken by either the mother or the father in order to take care of the child who is less than three years old, and do not exceed one year. Paid maternity leaves, not exceeding 90 days, are entitled to pregnant employees during their childbirth. This study, however, did not include paid maternity leave because it applies to only female employees. Another FFP is subsidized childcare cost. The purpose of this policy is to offer financial support in order to reduce the burden on their employees with childcare expenses such as daycare or kindergarten expenses. These four programs were selected because much empirical research has paid attention to them in the Western contexts (Grover and Crooker 1995).

In addition, South Korea has some different kinds of family supportive policies when compared to global ones. ‘Subsidized family-event cost’ is a special family-friendly benefit in South Korea. Employees receive partial financial support when they marry, when their family members pass away, or when they celebrate their parents’ 60th or 70th birthday. The partial payment of family events is based on the virtue of mutual help, which is a traditional value of South Korea (Oh et al. 1994). People influenced by Confucianism tend to distinguish in-group members from out-group members and treat the in-group more collaboratively and intimately than the out-group. Harmony, solidarity, and mutual help are emphasized (Oh et al. 1994), which are reflected in South Korean customs. For example, people give gift money or condolence money to neighbors who have celebrations (i.e., wedding, 1st, birthday of a child, 60th or 70th birthday, etc.) or sad occurrences (i.e., funerals). In addition to offering financial help, South Koreans offer community members their service and time (i.e., cooking, cleaning, and odd jobs).

Another FFP unique to South Korea is ‘support for housing’. South Korea has suffered from housing shortages since 1970s (Blankenship 2002). As the urban housing supply has not been able to keep up with the rapid rates of urbanization and industrialization, the supply of housing is relatively slow in urban areas (Son et al. 2003). Although the government has paid attention to resolving the scarcity of housing, the ratio of families owning their homes declined until 1990: 63.6% in 1975, 49.9% in 1990, and 53.3% in 1995 (Son et al. 2003). In addition, South Korea has a unique Chonsei lease system. The Korean real estate rental market is based on Chonsei lease contracts: tenants pays an upfront deposit instead of monthly rent payments and receive the deposit from their homeowners at the end of the usual two-year rental term. The deposit of Chonsei lease contract is estimated 40 to 80% of the value of property (Ambrose and Kim 2003). Until the early 1990s, housing prices rose faster than real income or general inflation, and the Chonsei rent increased much faster than the sale price for all housing types (Son et al. 2003). Given that tenants are less wealthy than homeowners, they may be affected by sudden changes, particularly rapid increases, in housing expenses. Tables 1 and 2 display the rapid rise of urban housing prices in South Korea from 2000 to 2007.

**Table 1:** **Changes (%)** ^**a**^ **in urban housing purchase price & Chonsei lease price over the last year**

Year	1999	2000	2001	2002	2003	2004	2005	2006	2007
Housing sale price	3.4	0.4	9.9	16.4	5.7	−2.1	4.0	11.6	3.1
Chonsei price	16.8	11.1	16.4	10.1	−1.4	−5.0	3.0	6.5	2.6

SOURCE: Kookmin Bank, Monthly Report Nationwide Housing Price Trend Survey, Each year.

^a)^ As of Dec. of each year.

**Table 2:** **Urban housing sale price indices & Chonsei price indices** ^**b**^ **(12.2008 = 100)**

Year	2000	2001	2002	2003	2004	2005	2006	2007	2008
Housing sale price	61.2	67.2	78.3	82.7	81.0	84.3	94.1	97.0	100.0
*Chonsei* price	72.8	84.8	93.9	91.9	87.4	90.1	95.9	98.3	100.0

SOURCE: Statistics Korea; ^b)^ As of Dec. of each year.

Moreover, companied with rising property prices, housing expenses are high (Son et al. 2003). Housing is an important issue to employees, especially married ones. By providing financial aid for housing such as mortgage loans, many companies respond to employees’ needs related to family. In general, married employees and single ones with a dependent family benefit from this program. Although this benefit is likely to be offered by employers with 1,000 or more employees, 47.9% of companies in South Korea provide financial aid for their employees’ housing costs (Lee 2009).

### Theoretical backgrounds and hypotheses

#### Family-friendly policies and job satisfaction

Job satisfaction is defined as “a general pleasurable or positive emotional state resulting from the appraisal of one’s job experiences” (Locke 1976: p. 1300). The literature has identified two factors which affect job satisfaction: 1) intrinsic factors such as chance for personal growth or promotion, achievement, and recognition; and 2) extrinsic factors such as pay, physical working conditions, and job security (Ghazzawi and Smith 2009: p. 301-302). Some researchers have reported that extrinsic factors are likely to decrease or have no effect on job satisfaction (e.g., Herzberg 1987,2003; Herzberg et al. 1959). However, other studies have found that extrinsic factors can be significant determinants of job satisfaction (e.g., Ghazzawi 2008; Kickham 2007). For example, job satisfaction or dissatisfaction is influenced by company policies such as flexible work schedule (Boehle et al. 2000; Yazel 2001). Brief (1998) reported that pay and benefits can play a significant role in job satisfaction. Given this, family-friendly policies may impact job satisfaction, although they are extrinsically, rather than intrinsically, related to job satisfaction.

According to organizational support theory, employee attitudes toward organizations are affected by the extent to which they meet their employees’ socioeconomic needs (Eisenberger et al. 1986). Based on favorable or unfavorable treatments provided by employers, employees tend to judge whether their employers favor or disfavor them (Rhoade and Eisenberger 2002: p. 698). Eisenberger et al.(1986) and Rhoade and Eisenberger (2002) stated that organizational support is largely categorized into two parts: 1) the extent to which the organization sets a high value on employees’ contributions; and 2) the extent to which it considers employees’ well-being (as cited in Dixmon et al. 2007: p. 238). Family-friendly policies can be related to the latter in that their purposes help employees to balance work and family, in turn contributing to employees’ well-being. In other words, employees may view family-responsive programs as indicators of organizational supportiveness, which in turn affects job satisfaction.

In this light, the literature on FFP has provided evidence of positive impact of FFP on job satisfaction (e.g., Chiu and Ng 1999; Frye and Breaugh 2004; Judge et al. 1994). Frye and Breugh (2004) found that family-friendly policies are negatively related to work-family conflict, in turn being positively correlated to job satisfaction. They also found a positive direct path from family-friendly policies to job satisfaction. Thompson et al. (1999) found that the availability of FFPs has negative effects on work-family conflict. Judge et al. (1994) reported that work-family policies are significantly positively associated with job satisfaction. Thus, I would like to hypothesize:

*Hypothesis* 1: The availability of family-friendly policies is positively related to job satisfaction in South Korea.

#### Family-friendly policies and organizational commitment

Social exchange theory and the norms of reciprocity have provided the theoretical backgrounds for the relationships between FFP and employee attitudes, specifically organizational commitment (Blau 1994; Eisenberger et al. 1986). Organizational commitment is defined as an “individual’s strong identification with the goals and values of the employing organization, involvement in an organizational role they perceive to be related to these goals and values” (Porter et al. 1974: p. 604). According to social exchange theory, employees tend to feel obligated to reciprocate when they are provided benefits (Prottas et al. 2007). In other words, employees may make themselves more committed to their current organizations in order to reciprocate benefits provided by their employer (Lambert 2000; Thompson et al. 2004). Employees may perceive that FFPs are indicators of organizational supportiveness.

Thus, as research has provided empirical evidence that HRM policies are responsive to employees’ needs, family-friendly programs have positive effects on organizational commitment (e.g., Bashir and Ramay 2008; Chiu and Ng 1999; Grover and Crooker 1995; Guy 1993). Similarly, Park and Kim (2001) showed that FFPs in South Korea are positively associated with job satisfaction and organizational commitment. Dockel’s study (Dockel A: The effect of retention factors on organizational commitment: an investigation of high technology employees, Unpublished) found that family-responsive policies are strongly and significantly associated with organizational commitment (as cited in Bashir and Ramay 2008: p. 228). Grover and Crooker (1995) and Haar and Spell (2004) suggested that the availability of FFP is positively associated with organizational commitment. FFPs are included in such HRM policies (Goldberg et al. 1989; Grover and Crooker 1995; Youngblood and Chmbers-Cook 1984; as cited in Chiu and Ng 1999: p. 490-491). Many studies have found that work-friendly benefits contribute to an increase in employees’ commitment because the benefits help to reduce family responsibilities (Grover and Crooker 1995; Halpern 2005; Kossek et al. 2001).

*Hypothesis 2*: The availability of family-friendly policies is positively related to organizational commitment in South Korea.

## Results and discussion

### Results

Means, standard deviations, and correlations were assessed for each variable. Table 3 displays a correlation matrix showing the means and standard deviations of the variables and the relationships among the variables. There were significant and positive correlations between the six family-friendly policies and job satisfaction (.12 ≤ *r* ≤ .2, *p* < .001) and organizational commitment (.18 ≤ *r* ≤ .25, *p* < .001), providing initial support for the hypotheses.

**Table 3:** **Basic statistics, reliabilities, & correlations**

Variable	Mean	SD	1	2	3	4	5	6	7	8	9	10	11	12	13	14
1. Job satisfaction	3.36	.65	(.92)													
2. Organizational commitment	3.17	.69	.72^**^	(.92)												
3. Paid leave	.44	.50	.20^**^	.25^**^	-											
4. Sick leave	.40	.49	.18^**^	.24^**^	.57^**^	-										
5. Childcare leave	.11	.31	.16^**^	.19^**^	.29^**^	.38^**^	-									
6. Partial payment of family events	.36	.48	.18^**^	.22^**^	.47^**^	.55^**^	.31^**^	-								
7. Subsidized childcare cost	.07	.25	.12^**^	.18^**^	.27^**^	.31^**^	.44^**^	.33^**^	-							
8. Support for housing	.10	.30	.17^**^	.23^**^	.31^**^	.37^**^	.40^**^	.42^**^	.54**	-						
9. Pay satisfaction	2.75	.75	.40**	.44**	.20**	.21**	.16**	.19**	-.17**	.20**	-					
10. Gender	.40	.50	.01	.02	-.15**	-.18**	-.02	-.18**	-.11**	-.14**	-.02	-				
11. Age	39.02	11.15	-.08**	-.10**	-.16**	-.21**	-.17**	-.16**	-.10**	-.09**	.09**	-.05**	-			
12. Education	.71	.91	.16**	.17**	.21**	.27**	.21**	.23**	.17**	.18**	-.15**	-.15**	-.36**	-		
13. Marital status	.64	.48	.02	.02	.04*	.04*	.01	.05**	.09**	.08**	-.04*	-.09*	.41**	-.11**	-	
14. Tenure	56.18	61.14	.10**	.13**	.20**	.24**	.15**	.26**	.24**	.30**	-.15**	-.18**	.25**	-.03	.23**	-

N for pay satisfaction = 2,993; N for tenure = 2,994; and other Ns = 2,995. Craonbach’s α in parenthesis.

** *p* < .01, * *p* < .05 (2-tailed).

Table 4 displays the percent of respondents who were entitled to apply to each FFP. The FFPs used in the current study can be divided into two types: 1) mandatory FFPs, which are legally protected such as paid leave, sick leave, and childcare leave; and 2) voluntary FFPs, which are discretionary benefits provided by organizations such as subsidized family event cost, childcare cost, and support for housing. Only 6.8% of the respondents could apply for subsidized childcare costs and only 10.2% could apply for housing support. In contrast, as more workers were eligible for mandatory FFPs (paid leave, sick leave, and childcare leave), relatively more respondents could apply for them than voluntary FFPs.

**Table 4:** **Percentage of FFPs Eligibility**

	FFPs	Eligible (%)	Not Eligible (%)	Total (%)
Mandatory FFPs (leave programs)	Paid leave	43.6	56.4	100
	Sick leave	40.4	59.6	100
	Childcare leave	10.5	89.5	100
Voluntary FFPs (economic support)	Subsidized family event cost	36.4	63.6	100
	Subsidized childcare cost	6.8	93.2	100
	Support for housing	10.2	89.8	100

To test the effects of the availability of FFPs on employee attitudes, a hierarchical regression analysis was employed using SPSS 17.0 software. Personal demographic variables (e.g., gender, age, education, etc.) were regressed on outcome variables (job satisfaction and organizational commitment) in the first step, and then the availability of FFPs was included in the second step. This analysis controlled for the relationships between personal demographics and employee attitude variables before testing the relationships between the availability of family-friendly programs and outcome variables. The increase in *R*^2^ from step 1 to step 2 indicated the effectiveness of the availability of FFPs. The outcomes of these analyses are presented in Tables 5 and 6.

**Table 5:** **Job satisfaction & FFPs: Hierarchical regression analysis**

Step	Variables	Beta	***R*** ^2^	***F***	∆***R*** ^2^	***F*** change
Dependent Variable: Job Satisfaction			.175	153.82***		
1	(Constant)	2.44**				
	Pay satisfaction	.38***				
	Gender	.02				
	Age	-.02				
	Education level	.11***				
2	(Constant)	2.4***	.194	69.42***	0.02	11.03***
	Pay satisfaction	.35***				
	Gender	.04***				
	Age	.001				
	Education	.08***				
	Paid leave	.08***				
	Sick leave	.01				
	Childcare leave	.04*				
	Subsidized family-event cost	.04†				
	Subsidized childcare cost	-.02				
	Support for housing	.05*				

† *p* < .10, * *p* < .05, ** *p* < .01, ***p < .001.

**Table 6:** **Organizational commitment & FFPs: Hierarchical regression analysis**

Step	Variables	Beta	***R*** ^2^	***F***	∆***R*** ^2^	***F*** change
Dependent Variable: Organizational Commitment						
1	(Constant)	2.3***	.05	47.41***		
	Tenure	.1.3***				
	Education level	.18***				
	Marital status	.01				
2	(Constant)	2.93***	.11	38.57***	.06	32.59***
	Tenure	.04*				
	Education level	.09***				
	Marital status	.003				
	Paid leave	.12***				
	Sick leave	.07**				
	Childcare leave	.05*				
	Subsidized family-event cost	.04†				
	Subsidized childcare cost	.01				
	Support for housing	.09***				

† *p* < .10, * *p* < .05, ** *p* < .01, *** *p* < .001.

Hypothesis 1 which states that the availability of FFPs would have positive effects on job satisfaction was partially supported. Controlling for gender, age, and level of education, the availability of FFPs contributed to increase in the percentage of variance explained in predicting job satisfaction (∆*R*^2^ = .018, *p* < .001). However, among the family-friendly policies, paid leave (*p* < .001), childcare leave (*p* = .04), and support for housing (*p* = .03) were statistically significant predictors of employees’ job satisfaction. The coefficients of those practices were small, which indicated that the three family-friendly practices had small effects on job satisfaction. Taken together, employees who were entitled to receive paid leave, childcare leave, and support for housing were likely to show slightly higher levels of job satisfaction than those who were not. In addition, mandatory FFPs were more likely to influence job satisfaction than voluntary FFPs. On the other hand, sick leave, subsidized family event costs, and subsidized childcare costs were not significant predictors of job satisfaction.

Hypothesis 2 was also partially supported. Regarding *R*^2^ change, an additional 6% of the variance in organizational commitment was explained by the availability of FFPs (*p* < .001). Among the family-friendly policies, paid leave (*p* < .001), sick leave (*p* = .006), childcare leave (*p* = .012), and support for housing (*p* < .001) were statistically significant predictors of employees’ organizational commitment. This means that employees who are eligible to apply for paid leave, sick leave, childcare leave, and support for housing are likely to show slightly higher levels of organizational commitment than those who are not . Like job satisfaction, mandatory FFPs were more likely to affect job satisfaction than voluntary FFPs. Also, subsidized family event cost was a marginally significant predictor of organizational commitment (*p* = .1). On the other hand, childcare costs did not statistically have an effect on organizational commitment. In other words, the availability of these three family-friendly programs would not contribute to predicting organizational commitment.

This study aimed to investigate the impact of FFP on employee attitudes in the private sector of South Korea. The findings of the current study provided partial support for the effects of family-friendly policies on job satisfaction and organizational commitment. Paid leave, childcare leave, and support for housing were significant predictors of both job satisfaction and organizational commitment. Moreover, subsidized family-event cost was a marginally significant predictor of the two employee attitudes, and sick leave was a valid predictor of organizational commitment, which was consistent with previous findings (e.g., Chiu and Ng 1999; Wang and Walumbwa 2007).

However, contrary to expectations, the positive relationships between subsidized childcare cost and both job satisfaction and organizational commitment were not supported. Support for childcare expenses is usually taken by young workers with a child who is less than three years old. Compared to other benefits, there are relatively few employees who benefit from the childcare expenses provided by the companies. Only 198 respondents, which are 6.8% of the sample, are eligible to apply for childcare expenses. This is relatively low, compared with the number of respondents who can apply for the other FFPs such as paid leave (1,268) and sick leave (1,174). If the relationships between childcare expenses and either job satisfaction or organizational commitment were investigated in organizations in which employees with young children work, different results may be reported.

The findings of this study suggest that paid leave, sick leave, support for housing, childcare leave, and subsidized family-event costs enhance employees’ positive attitudes. However, the effects of those policies on job satisfaction and organizational commitment were not great. This may be because this study investigated the relationships between the availability of family-friendly programs, rather than use of such programs, and attitudes. The respondents who were eligible for applying for these family supportive policies may not have taken advantage of them, and this in turn means that organizational commitment and job satisfaction were less affected by these particular FFPs (Thompson et al. 2004). Therefore, regarding FFPs in general, availability alone may not affect job satisfaction and commitment toward the organizations. Future research should be conducted on the relationships between use of family-friendly programs and job satisfaction and organizational commitment.

Although the hypotheses were only partially supported, the outcomes of the current study make contributions to the literature on FFP, job satisfaction, and organizational commitment. The research on the links between employee attitudes and family-friendly benefits has been heavily researched in the U.S. while few studies have been conducted in the private sector of South Korea. The findings of this study contribute to an understanding of the effects of FFPs on job satisfaction and organizational commitment by investigating the private sector of South Korea.

In addition, the literature on FFPs has mainly focused on paid leave, sick leave, childcare leave, and subsidized childcare costs (e.g., Grover and Crooker 1995). In addition to these policies, this research included various family-friendly policies such as partial payment of family event expenses and support for housing, which are unique family supportive programs in South Korea. Grounded in collectivism and Confucianism, South Korea has emphasized solidarity, cooperation, and mutual help. Subsidized family event cost reflects such values. Also, high housing expenses of South Korea contribute to the benefit of support for housing. With high housing prices, Cheonse lease contract, which is unique to South Korea, requires tenants to make a significant deposit. These contextual factors explain why employers provide the benefit of support for housing expenses. Workers with a dependent family, particularly married employees, are likely to benefit from this program.

The findings of this study imply that the literature on FFPs needs to be extended to the contexts outside of the U.S. Support for housing is positively related to both job satisfaction and organizational commitment. Also, partial payment of family events contributes to job satisfaction. As mentioned above, housing plays a critical role in the stabilization of livelihood in South Korea. Although around 10% of the sample can benefit from support for housing, it was positively related to both job satisfaction and organizational commitment. In this light, policies of support for housing may have positive effects on employee attitudes toward the organization, job, or both in South Korea. This implies that FFPs should address employees’ socioeconomic needs, which are grounded in the social context.

Additionally, KLIPS^b^ data were appropriate for the purpose of this study in that they included information regarding the availability of family-friendly policies and employee attitudes. Unlike studies based on a sample of women or married employees (e.g., Ahmad 1996; Samad 2006), heterogeneous groups of employees were included in KLIPS data: male, female, married, single, professionals, and so on. Family-friendly policies have become important issues, as the diversity in the organization has grown. This implies that an evaluation of the functionality of FFPs needs to be conducted in a variety of populations. Considering this, KLIPS data could be good sources of diverse groups, which in turn contributes to research on FFPs.

#### Limitations and future research

Despite these contributions, this study has some limitations. First, since this research used data collected at one point in time, it is hard to argue conclusively that the availability of FFPs were causally associated with job satisfaction, organizational commitment, or both. Therefore, future research needs to be conducted using longitudinal sample data.

In addition to job satisfaction and organizational commitment, future research needs to be conducted on the relationships between family supportive programs and employee attitudes such as job motivation and employee morale in the private sector of South Korea. In particular, job motivation has positive effects on work productivity (Ellickson and Logsdon 2002; Roberts 2002). However, little research has dealt with the impacts of FFPs on job motivation (Moon and Roh 2010) in South Korea.

Also, based on the findings of this study, future research may investigate organizational level outcomes such as extra-role behaviors (ERBs), turnover, or withdrawal behaviors. Satisfied employees are more likely to show high levels of ERBs than are unsatisfied employees because satisfaction is positively related to ERBs (e.g., Lee et al. 2006). Similarly, such employees are less likely to engage in unproductive behaviors such as turnover and withdrawals. Much research has reported that organizational commitment is a significant predictor of ERBs (e.g., Colquitt et al. 2001) and is negatively associated with turnover (Meyer and Allen 1997). The current study found that the availability of FFPs is positively related to organizational commitment. This suggests that FFPs might be related to employees’ conductive behaviors (i.e., ERBs) and deconstructive behaviors (i.e., turnover, withdrawal behaviors, etc.) towards their job or organization. In particular, ERBs and turnover have been considered important issues in organization research. Therefore, research on the link of FFPs to these organizational level outcomes would provide insights into the functionality of FFPs.

Another direction of future research might be to examine the relationships between organizational commitment and FFPs. Organizational commitment is categorized into three types: 1) continuance commitment; 2) affective commitment (Allen and Meyer 1990); and 3) normative commitment (Meyer and Herscovitch 2001). Research that explores the relationships between family-friendly policies and each dimension of commitment would contribute to the literature in that the effects of family supportive programs on organizational commitment could be elaborated.

Finally, future research needs to be conducted on comparing the effectiveness of the family-friendly policies grounded in benefits and that of flexibility of work schedule on employee well-being or attitudes in South Korea. The current study deals with the impacts of family-friendly benefits on employee attitudes. By including variables regarding flexibility, future research would provide managerial implications regarding which practices should be more weighted in implementing family-friendly policies.

## Conclusions

To sum up, family-friendly policies contribute to positive effects on employee attitudes. Although the effectiveness of those programs is not necessarily significant, this study provides support for the positive relationships between family-friendly programs and job satisfaction and organizational commitment in the private sector of South Korea. In particular, paid leave, childcare leave, and support for housing are positively related to both job satisfaction and organizational commitment. Future research should attempt to expand on this study by including variables relevant to family-friendly policies.

## Methods

### Sample and procedure

To test the hypotheses, this research used the 10th Wave of KLIPS, which was collected in 2007. KLIPS was approved by Statistics Korea, a national statics office of South Korea; and the approval number is 33601. KLIPS data are based on the nation-wide labor-related panel survey. Also, KLIPS data sets consist of two parts: the Individual dataset and the Household dataset. The current research is based on the respondents working in the private sector of the Individual dataset, which produces a cross-section of data which has been used for individual-level analysis. Although KLIPS data were not collected for this study, the Individual dataset includes information related to the current research: family-friendly benefits, demographics (age, gender, level of education, tenure, etc.), the status of economic activity, employment type, attitudes (i.e., job satisfaction, life satisfaction, and organizational commitment), company type and size, industry and occupation type, and so on. KLIPS data are collected in three ways: 1) a self-interview; 2) a proxy interview; and 3) a combination of both. In this study, only respondents via the self-interview were included in order to reduce errors that might exist with data collected from the other two types of interview.

In this study, I obtained a sample of 2,905 from the Individual dataset of KLIPS data, who were employees in the private sector. A demographic breakdown of respondents is as follows: 60.1% were male and 39.9% were female. The average age of the participants was 39 years (SD = 11.15). The average tenure in the companies was 56.18 months (SD = 61.13). 39.6% of the respondents had a high school diploma or lower; 18.4% had a 2-year college degree; 21.9% had a 4-year university degree; and 4% had a master’s degree or higher. Also, 28.3% were single; 63.8% were married; 3.8% were divorced; 1.2% were separated; and 3% had spouse who had passed away. With regard to employment type, 71% of the respondents were regular workers and 29% were irregular workers. In terms of industry, 31% were manufacturing; 15.2% were wholesale and retail trade; 12.3% were business services; 9.6% were construction; 7.1% were hotels and restaurants; 4.5% were transport; and education were 4%. Composition of occupation of the respondents is as follows: 15.5% were technician/associate professionals; 16.4% were business clerks; 17.6% were craft and related trades workers; 12.8% were plant/machine operators; 11.9% were elementary occupation; 9.2% were professionals; 7.5% were service workers; and 7.6% were sales workers. Table 7 presents demographics of the sample.

**Table 7:** **Demographic characteristics of sample**

Demography	***n***	%		***n***	%
*Age*			*Gender*		
20 or less	23	.8	Male	1,747	60.1
20s	631	21.7	Female	1,158	39.9
30s	983	33.8	*Marital status*		
40s	731	25.2	Single	821	28.3
50s	401	13.8	Married	1,852	63.8
60s or more	136	4.7	Separated	36	1.2
*Education level*			Divorced	110	3.8
High school or lower	1647	56.7	Spouse passed away	86	3.0
2-year college	535	18.4	*Employment Type*		
Bachelors	636	21.9	Regular worker	2,062	71.0
Masters or higher	87	3.0	Irregular worker	843	29.0
*Industry*			*Occupation*		
Wholesale & Retail trade	443	15.2	Technician/Associate professionals	449	15.5
Manufacturing	901	31	Clerk	447	16.4
Post & Telecommunication	48	1.7	Craft & Related trades workers	510	17.6
Education	116	4.0	Plant/Machine operators	373	12.8
Construction	278	9.6	Elementary occupation	345	11.9
Hotels & Restaurant	207	7.1	Professionals	226	9.2
Business services	356	12.3	Service workers	217	7.5
Health & Social work	102	3.5	Sale workers	222	7.6
Finance & Insurance	94	3.2	Others	46	1.5
Transport	131	4.5			
Others	319	7.9			

### Measures

#### The availability of family-friendly policies (FFPs)

I measured *the availability of FFPs* with the participants’ responses to the questions of whether an individual employee is entitled to receive family-friendly programs by his/her organization or employer. Six family-friendly programs were included in this study: paid leave, sick leave, childcare leave, partial payment of childcare expenses, partial payment of family events (marriage, funeral, etc.), and support for housing (mortgage loan, etc.). The availability of FFPs was assessed by surveying individuals with questions such as “Are you entitled to receive to paid leave?” and “Are you entitled to receive childcare expenses?” Respondents were asked to answer ‘yes’ if they can apply to a certain family-friendly program and ‘no’ if they cannot, or the respondent answered “I don’t know”. The responses were dummy coded into 0 = *no* and 1 = *yes*.

#### Organizational commitment

In KLIPS, *organizational commitment* was measured by responses to five items from the Organizational Commitment Questionnaire (OCQ) developed by Porter et al. (1974). Items of organizational commitment were as follows: ‘This is a good company to work at,’ ‘I am glad to have joined this company,’ ‘I would recommend joining this company to my friends who are searching for a job,’ ‘I take pride in being a part of this company,’ and ‘I hope to continue working at this company if other things remain the same.’ The ratings were on a five-point Likert scale ranging from 1 = *strongly disagree* to 5 = *strongly agree*. Organizational commitment yielded good reliability (Cronbach’s α = .92).

#### Job satisfaction

*Job satisfaction* of KLIPS was measured by responses to five items developed by Brayfield and Rothe (1951). The items were as follows: ‘I'm satisfied with the job I'm currently doing,’ ‘I'm glad to have joined this company,’ ‘I feel this job personally rewarding,’ ‘I enjoy this job,’ and ‘I want to continue this job if other things remain the same.’ The ratings were on a five-point Likert scale ranging from 1 = *strongly disagree* to 5 = *strongly agree*. Reliability test of job satisfaction yielded alpha coefficient of .92 (Cronbach’s α = .92).

#### Control variables

The literature has found that some demographic variables and wage satisfaction are associated with job satisfaction (e.g., Sagas and Cunningham 2005; Judge and Watanabe 1993). Gender (Joiner and Bakalis 2006; Sagas and Cunningham 2005), age, and education (Joiner and Bakalis 2006; Judge and Watanabe 1993) are related to job satisfaction. For example, female employees are likely to report lower levels of job satisfaction (Sagas and Cunningham 2005) than are male employees. As wage satisfaction is also positively associated with job satisfaction (Sousa-Poza 2000), it was included in the analysis as a control variable.

In addition, Mathieu and Zajac’s (1990) meta-analysis provided evidence that married employees are likely to exhibit slightly higher levels of organizational commitment than those of unmarried employees (Chiu & Ng 1999: p. 489). For instance, Joiner and Bakalis (2006) provided similar findings to those of Mathieu and Zajac’s study (1990). Also, tenure (Joiner and Bakalis 2006; Hrebiniak and Alutto 1972; Angle and Perry 1983; Mottaz 1988; Iverson and Buttigieg 1999), education level and marital status (Joiner and Bakalis 2006) have been shown to be related to organizational commitment. Joiner and Bakalis (2006) also found that tenure was negatively associated with commitment.

To sum up, this study included as control variables respondents’ age (in years); gender (0 = *male*, 1 = *female*); level of education (0 = *high school diploma or lower*, 1 = 2-*year college degree*, 2 = *4-year university degree*, 3 = *master’s degree or higher*); marital status (0 = *single*, 1 = *married*); and tenure (in months).

## Endnotes

^a^Available until October 25, 2013 at http://glaw.scourt.go.kr.

^b^KLIPS consists of cross-sectional data and time-series data. It has been conducted annually on a sample of 5,000 urban households and members of the households by the Korea Labor Institute since 1998. Questionnaires are administered on the 15+ year-old household members.

## Authors’ information

Dr. Kang’s research interests include expectations of extra-role behaviors, cross-cultural differences in organizational behavior, work-family issues, and so on.

## Abbreviations

FFP(s): Family-friendly policy (policies)
KLIPS: Korean labor and income panel study.
